# Utility of a next‐generation framework for assessment of genomic damage: A case study using the pharmaceutical drug candidate etoposide

**DOI:** 10.1002/em.22467

**Published:** 2021-11-22

**Authors:** John Nicolette, Mirjam Luijten, Jennifer C. Sasaki, Laura Custer, Michelle Embry, Roland Froetschl, George Johnson, Gladys Ouedraogo, Raja Settivari, Veronique Thybaud, Kerry L. Dearfield

**Affiliations:** ^1^ AbbVie, Inc. North Chicago Illinois USA; ^2^ Centre for Health Protection National Institute for Public Health and the Environment (RIVM) Bilthoven The Netherlands; ^3^ Seagen Inc. Bothell Washington USA; ^4^ Bristol‐Myers Squibb Company, Drug Safety Evaluation New Brunswick New Jersey USA; ^5^ Health and Environmental Sciences Institute Washington District of Columbia USA; ^6^ Federal Institute for Drugs and Medical Devices Bonn Germany; ^7^ Swansea University Medical School Swansea University Swansea UK; ^8^ L'Oréal Recherche & Innovation Aulnay‐Sous‐Bois France; ^9^ Corteva Agriscience Newark Delaware USA; ^10^ Sanofi, Research & Development Chilly‐Mazarin France; ^11^ Retired Burke Virginia USA

**Keywords:** etoposide, genetic toxicity, human health risk assessment, integrated testing strategy, mutagenicity

## Abstract

We present a hypothetical case study to examine the use of a next‐generation framework developed by the Genetic Toxicology Technical Committee of the Health and Environmental Sciences Institute for assessing the potential risk of genetic damage from a pharmaceutical perspective. We used etoposide, a genotoxic carcinogen, as a representative pharmaceutical for the purposes of this case study. Using the framework as guidance, we formulated a hypothetical scenario for the use of etoposide to illustrate the application of the framework to pharmaceuticals. We collected available data on etoposide considered relevant for assessment of genetic toxicity risk. From the data collected, we conducted a quantitative analysis to estimate margins of exposure (MOEs) to characterize the risk of genetic damage that could be used for decision‐making regarding the predefined hypothetical use. We found the framework useful for guiding the selection of appropriate tests and selecting relevant endpoints that reflected the potential for genetic damage in patients. The risk characterization, presented as MOEs, allows decision makers to discern how much benefit is critical to balance any adverse effect(s) that may be induced by the pharmaceutical. Interestingly, pharmaceutical development already incorporates several aspects of the framework per regulations and health authority expectations. Moreover, we observed that quality dose response data can be obtained with carefully planned but routinely conducted genetic toxicity testing. This case study demonstrates the utility of the next‐generation framework to quantitatively model human risk based on genetic damage, as applicable to pharmaceuticals.

## 1 INTRODUCTION

The testing of pharmaceutical drug candidates for the potential to cause genetic damage has been generally consistent for several decades. Pharmaceuticals are typically evaluated for the ability to cause gene mutations or chromosome damage in vitro prior to initiation of clinical trials in small groups of healthy volunteers or patients. In addition, in vivo tests in rodents for chromosome damage are required prior to larger clinical trials. These studies (the standard testing battery for pharmaceuticals) are required by regulatory guidelines (ICH, 2009a, 2011) and positive (genotoxic) pharmaceuticals in these tests are viewed to have the potential to increase cancer risk or cause genetic damage to germ cells. Conclusions from tests such as bacterial mutation (e.g., Ames test) and in vitro or in vivo chromosome aberrations or micronuclei are typically binary, with a yes/no outcome. Traditionally, these tests have been well‐suited to identify the hazard of possible genetic damage, particularly for direct DNA damaging agents. However, such a battery type of approach might not always be appropriate to detect the broad range of potential genetic damage.

As more has been learned about the structure–activity relationships and chemical motifs that impart genetic damage, medicinal chemists have learned to avoid chemical moieties that result in positive genetic toxicity results while maintaining desirable pharmaceutical properties. However, some pharmaceuticals, like etoposide, demonstrate an efficacious mechanism of action that derives clinical benefit via a genotoxic mode of action (e.g., binding to topoisomerase II), leading to double‐strand breaks (DSBs) (Caldecott et al., 1990).

Genetic damage has been established as a potential contributor to health issues/disease beyond cancer. Modes of action (MOAs) leading to genetic damage can play a key part in understanding disease progression and/or susceptibility (Dearfield et al., 2017). Beyond hazard identification, an understanding of the MOA and an assessment of genetic damage may allow for the identification of levels below which exposure to a chemical or pharmaceutical may pose an “acceptable” risk in relation to the expected benefits. Such levels could be estimated based on the so‐called genetic toxicology “point of departure” (PoD), to which uncertainty and safety factors are applied. Such new approaches for characterization of the broad range of potential genetic damage allow for identification and understanding of diverse MOAs, which may then be applied to risk assessment and regulatory decision‐making.

For genetic toxicology risk assessment, the next‐generation testing strategy developed by the Health and Environmental Sciences Institute (HESI) Genetic Toxicology Technical Committee (GTTC) involves a systematic and flexible approach for assessing the risk of genetic damage due to exposure to chemical substances (Dearfield et al., 2017). It places greater emphasis on estimating the potential risk of a substance if and when people are exposed rather than applying genetic toxicity testing data only for hazard identification.

The next‐generation testing strategy is a generic approach applicable to a wide range of chemicals. For the present case study, we use etoposide to evaluate the applicability of the strategy for a pharmaceutical, a chemical that is intended for human exposure. The GTTC recently published a similar case study using benzene to study the applicability of the approach for an industrial chemical (Luijten et al., 2020) where human exposures are unintentional. Per the benzene example, we used a retrospective approach for etoposide. Rather than generate a comprehensive literature review, the objective was to determine if the next‐generation risk assessment strategy could serve as a useful framework to collect required data for the identification and characterization of genotoxic hazard of a pharmaceutical. Hazard characterization included dose–response analysis and derivation of a PoD resulting from genetic damage which could lead to mutagenic and clastogenic outcomes.

## 2 ETOPOSIDE AS A CASE STUDY

The topoisomerase II inhibitor etoposide is an anticancer agent that has been evaluated in numerous genetic toxicity tests (IARC, 2000, 2012). Administration has been associated with secondary leukemia following therapeutic treatment (IARC, 2000, 2012). An adverse outcome pathway (AOP) describing infant leukemia resulting from chemical exposures was developed using the analogous etiology of secondary acute leukemia from etoposide therapy (Pelkonen et al., 2017). Given the wealth of readily available historical data, including studies representative of a pharmaceutical genotoxicity approval package, etoposide was selected as a case example to evaluate the applicability of the next‐generation testing strategy.

The next‐generation testing strategy provides a framework in which a series of steps allows for an evaluation of a chemical's genetic toxicity risk potential in a logical, and structured manner (Table 1). The steps first outline the problem formulation, including defining the exposed population, available information, and a description of the data needs to appropriately evaluate risk. Existing data are then assembled to build a knowledge base and create a biological argument for relevant testing. Once testing is performed and relevant datasets are identified, a quantitative analysis is performed to estimate the POD, which is then employed in a human risk assessment, resulting in risk characterization—a genetic toxicology risk assessment—for potentially exposed individuals. This assessment may then be used for risk management and regulatory decision‐making.

**TABLE 1:** Framework for next‐generation risk assessment^a^

Step	Process	Etoposide as case for adjuvant cancer therapy
1	Planning and scoping (incl. anticipated exposure)	Identify the relevant regulations in place for etoposide Determine the proposed clinical application(s) and the targeted patient population(s) Determine the most likely exposure route(s) for etoposide Determine how etoposide will be administered (alone or in combination with other drugs/therapies) Determine the category of anticipated exposure Begin risk/benefit analysis as patients will be purposely exposed to etoposide
2	Determine expected exposure	Determine expected pattern of exposure for etoposide therapy Estimate the projected efficacious level of etoposide exposure for the population group(s) of concern
3	Build knowledge base	Chemoinformatics: generate data using QSAR software tools; include predictions on possible metabolites Collect available data from relevant in vitro and in vivo toxicity studies Collect mechanistic information
4	Create rational biological argument	Based on the knowledge gathered, determine the potential of etoposide for induction of genetic damage. If so, determine the most likely mechanism(s) underlying this potential
5	Select assays and perform them	*Used published studies for etoposide (due to retrospective aspect of this case study)*
6	Review results	*Reviewed published studies for etoposide*
7	Select appropriate point of departure	Based on the rational biological argument identify relevant dataset(s) Conduct quantitative analyses to derive a PoD
8	Estimate acceptable levels for endpoints of human relevance	Determine whether it is appropriate to use a nonlinear approach Using the derived PoD determine the acceptable level of daily exposure for the population group(s) of concern
9	Risk characterization	Estimate the risk for humans by applying a MOE approach and comparing the exposure level to the acceptable level of daily exposure

^a^ Based on the framework described in Dearfield et al. (2017).

The retrospective analysis of etoposide literature data included standard genetic toxicology tests required for pharmaceutical development, per ICH S2R1 (ICH, 2011), as well as additional genotoxicity endpoints. Following the compilation of literature data, the next‐generation testing strategy framework (Table 1) was applied to a select set of studies. This stepwise, objective treatment of the data approximated a scenario of genetic toxicologists evaluating a novel small molecule pharmaceutical candidate during drug development. We demonstrate that application of the framework enabled consideration of test data beyond the standard ICH S2R1 pharmaceutical test battery (ICH, 2011). The appropriateness of the strategy was evaluated and modifications/improvements that could be applied for pharmaceutical candidates were noted. As this was a hypothetical demonstration using existing etoposide data intended to demonstrate the use of the framework, this work should not be construed as an endorsement for consideration for this drug for this indication as anticancer monotherapy.

### 2.1 Step 1: Planning and scoping (including anticipated exposure)

#### 2.1.1 Planning and scoping

The initial step in the application of the framework is problem scoping and planning of the work needed to inform management of any risk that might be associated with a drug. Problem formulation is the systematic process to guide and direct what *scientific* questions that must be addressed in the risk assessment (USEPA, 1998). Thus, the dataset collected may be different for the same chemical depending on the problem statement (e.g., different exposure scenarios). While planning and scoping outlines the broader questions including considerations such as logistics and costs, problem formulation focuses on the more specific scientific questions regarding the chemical's potential to cause genetic damage as well as exposure that may be relevant for human risk.

An important consideration for pharmaceuticals is that, in contrast to industrial chemicals where human exposure is generally unintended and without benefit, drugs are administered to derive a beneficial therapeutic effect. Drug exposure levels in patient populations would be expected to greatly surpass accidental environmental exposures, requiring consideration of the drug's MOA(s)/therapeutic target versus the potential to induce genetic alteration. Therefore, our evaluation needed to consider the MOAs based on the intended primary pharmacology of the drug.

Drugs are developed for specific therapeutic indications, typically with known targets/MOA. Etoposide was evaluated from the hypothetical perspective of a drug candidate under consideration for adjuvant therapy in curable cancers (see Table 1 for the risk management issues in this planning/scoping step). Consequently, the data were limited to retrospective evaluation that generally included select studies/assays that would be required for a first‐in‐human (FIH) clinical trial application. Acknowledging that pharmaceutical candidates being considered for advanced cancer therapies would not actually require genetic toxicology testing to advance to FIH clinical trials (ICH, 2009b), we made the hypothetical assumption that etoposide was under consideration for an indication less severe than advanced cancer—one where patients could be cured of their disease. This justified qualitative evaluation of the data to evaluate the risk benefit of such a treatment. To constrain the case study, additional literature‐cited mechanisms were considered out of scope for this case study.

#### 2.1.2 Scoping of anticipated exposure

The treatment population was assumed to be those on an adjuvant cancer therapy, or chemotherapy used after successful primary therapy (such as surgery) to reduce the risk of recurrence from cancer cells that may have escaped primary treatment. As adjuvant cancer therapies are expected to have low/negligible risk since the cancer/tumor would have already been removed or cured with the primary therapy, this low/negligible associated risk informed the extent of testing and review needed for a risk assessment of this exposed population. The focus on etoposide adjuvant cancer therapy assumed that exposures were limited to the subset of patients who could derive benefit. While the framework could encompass wide exposure to consumers, focusing this analysis to a specific disease scenario and specific patient population receiving the therapy restricted the anticipated exposure concern.

As the exposure scenario was frontline chemotherapy (e.g., primary therapy such as surgery, followed by the adjuvant therapy of etoposide), and not a second‐line therapy, the complication of previous exposures to additional genotoxic chemotherapies did not have to be addressed. To avoid complicating variables for this analysis, we evaluated etoposide as monotherapy (etoposide alone would be administered) in the adjuvant setting, although etoposide has commonly been used in combination therapy.

### 2.2 Step 2: Determination of expected exposure

The exposure assessment to a pharmaceutical can encompass a variety of target populations. Healthcare workers, manufacturing employees and the general population may be *unintentionally* exposed to a drug. In contrast to industrial chemicals (Luijten et al., 2020), patients are intentionally exposed to pharmaceutical therapies to alleviate disease. Unintentional exposure groups may be exposed for brief periods at low levels through daily work or environmental contamination. The biologic effects from such exposures provide no benefit.

While many chemicals are studied for potential toxicities to derive safe worker exposure limits or water quality standards, pharmaceuticals are relatively unique in that human exposure scenarios are extensively studied, via analysis of drug exposure levels following specific dose administration schedules and routes in animals, healthy volunteers and/or patients. Such data allow for more precise exposure assessment to determine the conditions in which efficacy is observed. These levels could be compared to exposures in experimental systems where genotoxicity occurs. The assumed target population, identified in the planning and scoping, are individuals with a high cancer cure rate. Exposure to agents that cause genetic damage in this patient cohort may therefore represent a different risk compared to other patient groups, as would be reflected in the risk characterization.

In adults, a typical monotherapy etoposide dosing regimen as primary treatment can range from 35–200 mg/m^2^ daily for up to 5 days (Cancer Care Ontario, 2020; Medscape, 2021). For use as an adjuvant treatment, a typically cited dose is 100 mg/m^2^, usually in combination with another chemotherapeutic (McHugh & Feldman, 2018; Motzer et al., 2000).

Etoposide can be administered orally or intravenously, with either short or long infusions for a variety of cancers (IARC, 2000; McLeod, 1997). Via the oral route, bioavailability is about 50% (IARC, 2000). Plasma protein binding is about 95% (IARC, 2000). In general, exposure is linear with increasing dose (Würthwein & Boos, 2002). The framework encourages collection of absorption, distribution, metabolism, and excretion (ADME) data for exposures. Unlike industrial chemicals, ADME properties of drugs are comprehensively evaluated during drug development. But in this case study, for simplicity, we assumed a maximum exposure scenario with an intravenous dosing regimen, resulting in 100% bioavailability, with distribution to most tissues in the body, eliminating the need to consider what fraction of dose is systemically available to cause the observed genetic toxicity.

### 2.3 Steps 3–4: Building the knowledge base and creation of a rational biological argument for genetic damage

#### 2.3.1 Building the knowledge base

The next steps in the framework involve the development of a knowledge base by collection and consolidation of existing data, and the creation of a biological argument (Table 1, Steps 5–6). Due to the retrospective nature of the case study, the knowledge base was developed in conjunction with a review of compiled in vitro and in vivo genotoxicity studies. During drug development, standard preclinical in vitro and in vivo genotoxicity test “battery” assays (ICH, 2011) are a reasonable start for developing the knowledge base. In addition, in silico examination of other drugs focusing on the same or similar therapeutic target/MOA is considered extremely useful for creating a biological argument and for identifying any needed additional tests.

#### 2.3.2 Target assessment/assessment of anticipated MOA

Etoposide antagonizes cell division and inhibits tumor growth, forming a complex with topoisomerase II and DNA. This complex formation is covalent and not reversible, preventing re‐ligation of the cleaved DNA double‐strand and leading to DSBs (Caldecott et al., 1990). The increase of DSBs will trigger DNA damage response via yH2AX signaling of DSB and activation of p53. DSB repair, namely homologous and non‐homologous end joining (NHEJ), will cope with the damage (de Campos‐Nebel et al., 2010; Malik et al., 2006; Smart et al., 2008; Sung et al., 2006). Exhausting repair capacity and damage accumulation ultimately leads to activation of apoptosis and cell death. Rapidly dividing cells like cancer cells will be more sensitive to this activity than resting cells, resulting in tumor cell death. However, organs containing rapidly dividing cells such bone marrow may also suffer. Chronic toxicity may also result from error prone DNA repair like NHEJ and accumulation of mutations resulting in increased cellular senescence and impaired cellular function in surviving cells. A preliminary AOP describing how topoisomerase II inhibition leads to increases in chromosome breaks and rearrangements and/or gene mutations is described in Sasaki et al. (2020).

#### 2.3.3 Creation of a rational biological argument for testing

Based on the intended pharmacological activity of etoposide and the preliminary AOP, testing that allows for assessment of chromosomal breakage events would be most valuable. Further relevant tests include genotoxicity studies detecting the relevant DNA damage, in this case DSBs, and studies which measure any key events for genetic damage, such as the γH2AX assay, in vivo complex of enzyme assays measuring DNA‐topoisomerase II covalent binding, supercoiled DNA cleavage/ relaxation assays and decatenation assay (Nitiss et al., 2012; Sahai & Kaplan, 1986).

### 2.4 Steps 5–6: Selecting and performing assays; reviewing results

Since etoposide is an extensively studied drug, this exercise examined selected studies that have been performed and were deemed appropriate to test the possible genetic adverse effects resulting from therapeutic anticancer treatment. The results of these studies were subsequently reviewed to identify those studies that offered the most relevant information to characterize the genotoxic potential of etoposide. Appropriate positive genotoxicity results, that would typically be generated in a drug development setting, were selected to identify a PoD for a quantitation of any possible risk to exposed patients, outside of its intended therapeutic use.

It should be clarified that to demonstrate the use of the framework, it was easier to use an example drug with plenty of existing data. However, the amount of data found is much more than would typically be generated for a drug candidate, and thus only select studies were chosen for consideration for quantitative analysis. Similarly, this work is not meant to represent a review of all genotoxicity and mechanistic research available for etoposide.

#### 2.4.1 In silico results: (Q)SAR and read‐across evaluations

Generally, there is little human data available for a pharmaceutical early in the development of a class of drugs. An in silico examination of a similar marketed product could provide insights into the likelihood of genetic damage from exposure to a novel drug of the same class. Using such in silico techniques are now commonplace in drug discovery and development, providing early insights into possible adverse effects. This in silico exercise examined etoposide as a novel candidate drug in several models.

(Q)SAR ([Quantitative] structure–activity relationship) models can identify structural features associated with various toxic effects including genotoxic potential. “Read‐across” is another useful tool to identify potential toxicities. This approach relies on structural analogs with experimental data to extrapolate potential genotoxic liabilities for an untested compound. These types of assessments are often conducted to support early drug development prior to conducting pivotal animal studies. Both chemists and toxicologists can utilize these tools to help establish an optimal testing strategy.

The potential genotoxicity of etoposide was evaluated in two commonly used models (Table 2). A review of the (Q)SAR data indicated that etoposide was part of the model training sets, highlighting the difficulty in retrospectively applying (Q)SAR models to analyze a known genotoxicant. Though etoposide was found in the training sets, we conducted the (Q)SAR analysis as it would have been done if etoposide was an “unknown” to the training set.

**TABLE 2:** Commonly used models for in silico (Q)SAR evaluation

Software	Model	Endpoint and prediction
Derek Nexus (version 6.01, 2020; Lhasa Limited, Leeds, UK)	Expert/rule‐based	In vitro mutagenicity—bacterial: negative (contains misclassified features)^a^ mutagenicity—mammalian: positive chromosomal damage: positive^a^ In vivo mutagenicity: negative chromosomal damage: negative
Leadscope Model Applier (version 2.4.1, 2020; Leadscope, Columbus, OH, USA)	Expert/rule‐based statistics‐based	In vitro mutagenicity—bacterial: negative^a^ In vitro mutagenicity *Salmonella*: negative^a^ mutagenicity—*Escherichia coli*/TA 102 (A‐T): positive^a^ clastogenicity: positive^a^ In vivo clastogenicity: positive^a^

^a^ Etoposide identified in the test reference set.

Etoposide was predicted negative for bacterial mutagenicity in most Ames strains but likely positive with *Escherichia coli* or *Salmonella typhimurium* strain TA102, and predicted positive for in vitro mutation in mammalian cells and for clastogenicity. The two models used differed in their prediction for in vivo clastogenicity.

In addition to performing (Q)SAR analysis for etoposide, a structural analog search was conducted. Teniposide, another topoisomerase II inhibitor with a known genetic toxicity profile, was identified. Overall, the interpretation of teniposide (Q)SAR predictions (not alerting for mutagenesis, alerting for clastogenesis in vitro and in vivo; possible DNA intercalation) and teniposide genetic toxicity data, along with our informed analysis of the MOA, indicated that etoposide could present a genotoxic risk through gene mutations or chromosome damage, to patients with potentially chronic or curable cancers. Based on the intended MOA for therapeutic use and the indications from the in silico results, clastogenicity is a very likely primary mechanism for etoposide's risk.

#### 2.4.2 In vitro results

After identifying genotoxicity flags in in silico systems, our assumed scenario of an adjuvant oncology therapy indication would typically be followed by a series of routine in vitro genetic toxicity tests (usually, a gene mutation test in bacteria and a chromosome damage test in cultured mammalian cells). Regulatory guidelines identified in the planning phase of the framework required these tests regardless of the (Q)SAR and read‐across findings for nonlife‐threatening indications (ICH, 2009a, 2011). In addition, while the step “Creation of a Rational Biological Argument for Testing” identified chromosome damage tests as pertinent to etoposide based on its MOA, these regulatory guidelines generally require testing for gene mutations prior to administering drugs to clinical trial subjects. A summary of the data from the reviewed in vitro genetic toxicology tests are depicted in Table 3.

**TABLE 3:** Summary of in vitro genetic toxicity testing of etoposide

Endpoint	Results	References
Gene mutation in bacteria and mammalian cells	Inconsistent results in bacterial reverse mutation test	Ashby et al. (1994), Gupta et al. (1987), Matney et al. (1985), Nakanomyo et al. (1986)
	Negative for gene mutations in mammalian cells	David et al. (2018)
	Positive for gene mutations in mammalian cells	Chatterjee et al. (1990), Ashby et al. (1994), Berger et al. (1996), David et al. (2018)
DNA damage	Single and double strand breaks in DNA in L1210 cells	Wozniak and Ross (1983)
Chromosome damage	Chromosomal aberrations, aneuploidy, SCE in CHO, L5178Y, and human cells (e.g., TK6, HepG2 cells)	Long et al. (1986), Tominaga et al. (1986), Kerrigan et al. (1987), Pommier et al. (1988), Lock and Ross (1990), Maraschin et al. (1990), Berger et al. (1991)
	Significant increase in micronuclei in rat cultured seminiferous tubules	Sjöblam et al. (1994), Slob, 2002)

In bacterial reverse mutation tests, etoposide treatment resulted in negative or weak‐positive gene mutation responses in *S. typhimurium* or *E. coli* in the presence or absence of an exogenous metabolic activation system (Ashby et al., 1994; Gupta et al., 1987; Matney et al., 1985; Nakanomyo et al., 1986). In mammalian cell mutation tests, etoposide increased gene mutation frequency in Chinese hamster ovary (CHO) and human T‐lymphoid cells (larger deletions) at the hypoxanthine‐guanine phosphoribosyl transferase (*HPRT*) and adenosine kinase *(ADK)* loci, and induced DNA strand breaks and sister‐chromatid exchanges (Chen et al., 1996; Gupta et al., 1987). Increased gene mutations observed at the thymidine kinase locus of L5178Y cells were mostly small colony mutants, indicating clastogenic origin (Ashby et al., 1994). In a recent in vitro study for mutation in the phosphatidylinositol glycan class A (*Pig‐a*) gene, etoposide did not increase the mutation frequency (% of GPI[−] cells) in L5178Y mouse lymphoma cells (confirmed using Sanger sequencing), suggesting that gene mutation mechanisms are not the primary MOA for etoposide under in vitro conditions (David et al., 2018).

Etoposide has clastogenic properties in a number of in vitro studies (CHO, L5178Y, HepG2, TK6 cells) at concentrations ranging from 40 ng/ml (Ashby et al., 1994; Boos & Stopper, 2000; Doherty et al., 2011; Fellows et al., 2008; Hermine et al., 1997; Larripa et al., 1992; Lynch et al., 2003; Thougaard et al., 2014; Westerink et al., 2011; Diaz et al., 2007; Smart et al., 2008; Tilmant et al., 2013). Human cells are more sensitive to etoposide compared to mouse cell lines in in vitro micronucleus tests and apoptosis studies (Laingam et al., 2008). The micronuclei induced by etoposide were shown to be a result of both clastogenic (61%–84% were kinetochore‐negative) and aneugenic (26%–39% were kinetochore‐positive) activity in CHO cell lines (V79‐4, irs‐1, and irs‐3) (Hermine et al., 1997).

Overall, in vitro testing indicated that etoposide has the potential to cause chromosomal damage, and while some individual tests showed positive results, the weight of evidence shows etoposide is less likely to cause gene mutations.

#### 2.4.3 In vivo results

A summary of the responses from the in vivo genetic toxicology tests reviewed is shown in Table 4. Consistent with in vitro results, etoposide induced a small but nonsignificant increase in *Tk*, but not *Hprt* mutation frequency in mice at doses up to 10 mg/kg (Dobrovolsky et al., 2002; Turner et al., 2001). As per the Transgenic Rodent Assay Information (TRAiD) database, etoposide yielded negative results in bone marrow, liver, and lung and when dosed at 25 mg/kg/d for 8–12 weeks via the intraperitoneal route in the mouse in transgenic (*lacZ*; i.e., Muta™Mouse and *lacZ* plasmid mouse) rodent mutation assay (Lambert et al., 2005). It should be noted, that the TRAiD database contains a majority of experiments which were conducted in the 1990s and early 2000s, before the standardization of the OECD TG488 test guideline (OECD, 2020). Consistently, in Sprague–Dawley rats, following a single intravenous dose of 5, 10, or 20 mg/kg, etoposide was negative in bone marrow *Pig‐a* and PIG reticulocyte (PIGRET) assays (Kimoto et al., 2016).

**TABLE 4:** Summary of in vivo nonclinical genotoxicity testing of etoposide

Assay (species/strain)	Results	Dose level, route, duration	Reference
**Gene mutation** *Inconsistent and negative results*			
TK^+/−^ male and female mice (C57BL/6 background)	No statistically significant increase in *Hprt* or *Tk* mutant frequency	1 and 5 mg/kg i.p., single dose	Dobrovolsky et al. (2002)
B62DF1 mice and APRT heterozygous mice	No statistically significant increase in *Hprt* mutant frequency. Significant increase in *Aprt* at 1 mg/kg, but not at 10 mg/kg. FISH analysis suggested mitotic recombination or chromosome loss and duplication as the mechanism of loss of heterozygosity in *Aprt* clones	1 or 10 mg/kg mg/kg i.p., single dose	Turner et al. (2001)
*lacZ* plasmid mouse Muta™ Mouse	Negative Negative	125 mg/kg i.p., five applications, sampling up to 35 days 1 mg/kg i.p., single dose, sampling on Day 14	Lambert et al. (2005), Tinwell et al. (1998)
*Pig‐a*, PIGRET (Male Sprague Dawley rat)	No statistically significant increase in mutant frequency	5, 10, and 20 mg/kg orally, single dose	Yamamoto and Wakata (2016)
**DNA damage**			
Alkaline Comet assay (Male Sprague Dawley rat)	Significant increases in mean tail moments at 1 and 4 h	5 and 50 mg/kg i.p., single dose	Godard et al. (1999)
B62DF1 mice	Significant increase in comet tail moments at 1 h	1 or 100 mg/kg mg/kg i.p., single dose	Turner et al. (2001)
Male Long–Evans rats	Significant increases in comet tail moments at 1.5 h, but not at 3 h	25 mg/kg, gavage, single dose	Spronck and Kirkland (2002)
**Chromosome damage**			
Swiss albino mouse	Significant increase in clastogenicity at 6 and 12 h	5, 10, 15, and 20 mg/kg .p., single dose	Agarwal et al. (1994)
B62DF1 mice	Significant increase from 0.1 to 1 mg/kg	0.1 to 16 mg/kg i.p., single dose; 24 h exposure	Turner et al. (2001)
MNT (male (102/ElxC3H/El) F1 mice) and FISH analysis	Significant increases in clastogenic and aneugenic responses	1 mg/kg i.p., single dose	Attia et al. (2003)
Male and female Swiss albino mice	Significant increases in chromosomal aberration in females and males at 20 mg/kg at 24 h Significant increases in in vivo MNT in males and females at 15 and 20 mg/kg at 30 h	10, 15, or 20 mg/kg i.p., single dose	Choudhury et al. (2004)
CD‐1 mice	Significant increases in in vivo MNT	0.75 to 6 mg/kg	Nakanomyo et al. (1986)
CD‐1 mice	Significant increases in in vivo MNT mainly due to whole chromosome lagging in spermatids at 24 h	25 mg/kg i.p., single dose	Kallio and Lähdetie (1993)
Male Sprague–Dawley rats	Significant increases in MNT in bone marrow and peripheral blood at all doses	14.3, 28.5, 57, and 114 mg/kg, gavage, 2 days	Fiedler et al. (2010)
Male and female F344 rats	Significant increases in in vivo MNT	1.14, 11.36, and 57 mg/kg, gavage, 14 days	Garriott et al. (1995)
Male Long–Evans rats	Significant increases in in vivo MNT	1 mg/kg, gavage, single dose	Spronck and Kirkland (2002)
Wistar rats [Crl:WI (Glx/Brl/Han)]	Significant increases in in vivo MNT only at 25 mg/kg	12.5 and 25 mg/kg, gavage, four doses	Tilmant et al. (2013)

Etoposide was shown to induce DNA damage in in vivo comet studies. In male B6D2F1 mice, etoposide increased DNA damage at dose levels up to 50 mg/kg (Turner et al., 2001). Similarly, in male Sprague–Dawley rats, at 5 and/or 50 mg/kg following a single dose, etoposide induced a positive response in the comet assay in whole blood, bone marrow, liver, and intestine at the 1‐h timepoint, and in bone marrow and intestine at the 4‐h timepoint. In contrast, kidney and thymus were negative in this assay (Godard et al., 1999; Kirkland et al., 2019).

In in vivo cytogenetic studies, etoposide has been shown to be a clastogen at high dose levels, as well as at lower, clinically relevant exposures. At doses from 5 to 20 mg/kg etoposide induced a dose‐dependent, significant increase in bone marrow clastogenicity in Swiss albino mice when administered via intraperitoneal administration (Agarwal et al., 1994; Choudhury et al., 2004). Similarly, doses from 0.5 to 10 mg/kg induced a dose‐dependent increase in sister chromatid exchanges. An increase in cell cycle time, as measured by average generation time, was also observed, with cells in the S‐phase being specifically targeted and blocked in the late S/G2 transition (Agarwal et al., 1994). In a subsequent single dose study at clinically relevant exposures, etoposide induced a significantly higher number of chromosomal aberrations and micronuclei in the bone marrow of male and female mice at the highest dose (20 mg/kg) tested (Choudhury et al., 2004). In the Fisher 344 rat, Garriott and coworkers reported a positive micronucleus response at 57 mg/kg (5x the clinical dose, on an equivalent mg/kg basis), following 14 days of repeat dose treatment (Garriott et al., 1995). In both the mouse and rat, following a single dose (mouse) or two repeat daily doses (rat) of etoposide, micronuclei were observed in spermatids at the diplotene diakinesis, late pachytene, preleptotene, and diplotene‐diakinesis stages (Kallio & Lähdetie, 1993, 1997; Lähdetie et al., 1994). In addition to somatic cell clastogenic effects, etoposide has been shown to induce aneuploidy and cell cycle arrest in male Balb/c mouse germ cells. Following a single intraperitoneal dose of 20 mg/kg etoposide, a statistically significant increase in CREST positive micronuclei were observed (Kallio & Lähdetie, 1997).

Taken together, the reviewed studies (Tables 3 and 4) suggest that the primary genotoxic mechanism for etoposide is chromosome damage. None of the reviewed studies noted drug exposure measurements, therefore, exposure‐response assessment data could not be evaluated.

#### 2.4.4 Review of results

While Tables 3 and 4 represent a summary of research that has been conducted with etoposide for evaluation of genotoxicity, not all of these tests would be necessary during drug development. Thus, bacterial reverse mutation tests may indicate the need for an in vivo gene mutation, such as the *lacZ* studies in transgenic rodents. Once determined as negative, no further in vivo gene mutation assays would be conducted. After obtaining positive results in an in vitro chromosomal aberration test, in vivo micronucleus testing would be conducted, usually in one species of rodent. A typical testing workflow for genetic toxicology support for a pharmaceutical and follow‐up testing needed based on the genotoxicity of etoposide is shown in Table 5.

**TABLE 5:** Typical genetic toxicology assessment for pharmaceuticals to support clinical trials, using select etoposide outcomes

Assessment	Outcome for etoposide	Follow‐up testing
In silico evaluation	possible gene mutagen, possible chromosome damage	Conduct Ames test; in vitro chromosome damage (aberrations or micronucleus test)
In vitro Ames	positive results	conduct in vivo gene mutation (Pig‐a or transgenic gene mutation)
In vivo *lacZ* mouse test	negative^a^	No mutation risk
In vitro Chromosome Aberrations	positive	conduct in vivo micronucleus and comet test
In vivo MN comet	positive	risk of chromosome damage

a Tissues studied: bone marrow, liver, and lung (Lambert et al., 2005).

Studies shown in Table 5 may be the only assay types needed to determine that etoposide causes chromosome damage and with an appropriately designed study, a dose–response analysis can be conducted to establish a PoD for this endpoint. With this latter summary in mind, analysis of the etoposide data set was progressed to Steps 7–9 of the framework.

### 2.5 Steps 7–8: Selecting an appropriate PoD and estimating acceptable levels for endpoints of human relevance

From the reviewed studies, the in vivo rat study described in Garriott et al. was selected for quantitative analysis as it demonstrated positive results in a bone marrow micronucleus assay (an appropriate assay given the known MOA of etoposide) (Garriott et al., 1995). The study also provided appropriate data to model the dose–response relationship in an in vivo system. A PoD for estimating a possible “safe” dose for a healthy clinical subject can be determined from the dose–response analysis. The selection such a dose for clinical trials would be a primary objective for the FIH trial for an adjunct oncology indication. With such an estimated dose, the probability of adverse genetic toxicity is by design reduced as much as possible while ensuring the safety of the therapy with an efficacious treatment.

#### 2.5.1 Quantitative analyses

**FIGURE 1:**
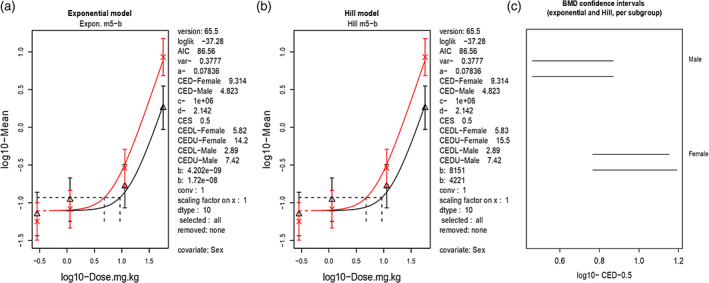
Covariate BMD analysis of %MN PCE in male (red line, cross character) and female (black line triangle character) rats from Garriott et al. (1995), using a CES of 50% (a,b). Results are shown for the exponential and Hill models. The BMDL–BMDU plot from the exponential (top line) and Hill (bottom line) model are also presented (c). Log10 used for each axis

The studies described by Garriott et al. (1995) using male and female F344 rats were analyzed (Table S1). The study design is representative of a typical pharmaceutical study that includes a micronucleus endpoint on a 2‐week general toxicity study, as well as providing dose–response data of low, medium, and high responses of micronucleus formation. These datasets were modeled using Benchmark Dose (BMD) analysis in PROAST (PROAST; www.rivm.nl/proast; Slob, 2002). We applied the covariate approach, where the model is fitted to a combination of datasets with dataset as a covariate, to improve the precision in the estimated BMDs (reflected by the width of the BMD confidence interval (CI) (Slob & Setzer, 2014; Wills et al., 2016). Models with additional parameters are only accepted if the difference in log‐likelihood exceeds the critical value at *p* <  0.05 (Slob & Setzer, 2014). In this way, it can be established which model parameters need to be estimated for each subgroup, and which parameters may be considered as constant among the subgroups of a combined data set. In general, it was assumed that the maximum response (parameter c) and log‐steepness (parameter d) (i.e., the two shape parameters) were equal for all response curves, while parameters for background response (parameter a), potency (parameter b) and var (i.e., within group variation) were examined for being covariate dependent (Wills et al., 2016). Application of the covariate BMD analysis showed that the dose responses for males and females were highly similar, which was reflected in the overlapping BMD CIs (Figure 1, Table 6). Using a critical effect size (CES) of 50%, as recommended for in vivo micronucleus data (Zeller et al., 2018) instead of the default value of 10% (EFSA, 2005), resulted in BMDL (i.e., the lower 90% CI of the benchmark dose) values of 2.89 and 5.82 mg/kg body weight for males and females, respectively (Table 6).

**TABLE 6:** Covariate BMD analysis using a CES of 50% was carried out using PROAST v65.5

BMD confidence interval bounds	MN PCE%	MN PCE%
	Male	Female
*BMDL*_*50*_ (mg/kg) (CES 50%)	2.89	5.82
*BMDU*_*50*_ (mg/kg) (CES 50%)	7.42	15.5

*Note*: Dose response data from the MN PCE% in male and female rats was assessed from the Garriot 1995 publication (Garriott et al., 1995). The lowest BMDL and highest BMDU from the Hill and exponential models (Figure 1) are presented. Abbreviations: BMDL, lower confidence limit of BMD; BMDU, upper confidence limit of BMD.

A similar analysis was conducted on data from a study reported by Fiedler et al. (2010), in which etoposide was tested in male Sprague–Dawley rats. In this study, the response for MN% only covered the medium and higher range, but not the low or very high response regions (Table S2). Consequently, the resulting BMD CIs were not as narrow as those obtained using the Garriott data, as shown by higher BMD CI ratios (Figure S1 and Table S3). Therefore, we considered the Garriott study more appropriate for our purposes.

##### Estimated acceptable levels for endpoints of human relevance

Based on the existing data and analyses, a BMDL_50_ of 2.89 mg/kg/day derived for rodents was an appropriate PoD to use as starting point for estimating allowable human exposure levels. Several methods exist for deriving a safe exposure dosage from animal data. In some cases, the PoD can be used in an equation such as seen in ICH Q3C for residual solvents which includes additional adjustment factors for determining a safe dose for human exposure such as adjusting for the test species, the duration of the study used in the analysis and the severity of the toxicity seen (ICH, 2019). This approach is often used with exposures to non‐mutagenic carcinogens as impurities in pharmaceuticals (Bercu et al., 2018).

For clinical development, the starting dose is determined by the regulatory guideline “Estimating the Maximum Safe Starting Dose in Initial Clinical Trials for Therapeutics in Adult Healthy Volunteers” (USFDA, 2005) as well as within ICH Guidance (ICH, 2009a). According to this guideline, the PoD, which could be a no adverse effect level (NOAEL) in mg/kg or mg/m^2^, is converted to an equivalent human dose by converting the animal dosage to equivalent dose based on body surface area. To apply this conversion, Table 1 of the guideline shows that we can first convert the rat PoD in mg/kg to a human equivalent mg/kg by dividing by 6.2 (or multiplying by 0.16): 2.89 mg/kg in rat / 6.2 = 0.47 mg/kg human equivalent dose.

Often for cancer drugs like etoposide, human dosages are expressed in mg/m^2^. Therefore, the human equivalent PoD in mg/kg could be converted to mg/m^2^. This is done by multiplying by the Km which converts mg/kg to mg/m^2^ for each species shown in Table 1 of the guideline. For humans, the Km is 37, thus:

0.47 mg/kg × 37 = 17.4 mg/m^2^ (USFDA, 2005).

While micronucleus induction may be monitored clinically, the assumption is that the effects from genetic damage manifest in such an assessment would be adverse and irreversible, increasing the risk to patients. Additional factors may contribute to the derivation of a starting dose. The US FDA guidance provides a method calculating a safe starting dose for healthy clinical subject, where our case example would be applied to patients who have curable cancer. Some leeway may be given under this situation to increase the starting dose level. Likewise, the dosing schedule may influence the risk characterization. If the adjuvant therapy were given less frequently than daily (for example once a week or once every other week) the daily burden from exposure to the genotoxic drug may be reduced such that a higher starting dose may be acceptable. As the dose increases during clinical trials, it may exceed the dosage where genetic damage would occur. Thus, the risk from the dose range expected to be administered along with other considerations needs to be considered in relation to the expected efficacious doses to determine if advancing to the clinic is feasible.

### 2.6 Step 9: Risk characterization

To characterize the risk toxicities of a pharmaceutical to humans, the expected human exposure is compared to the exposure or projected exposures that causes the hazard in each treatment condition. Risk characterization for pharmaceuticals considers additional factors, such as the benefit the exposure may provide the patient.

As the established clinical use of etoposide is treatment and cure of existing cancer, there is a positive risk–benefit ratio associated with therapeutic use of this drug. In contrast, use of etoposide alone in an adjuvant setting would be dependent on whether this drug could be shown to decrease the risk of recurrent cancer at dose levels associated with very limited or negligible genotoxic risk. Clinical data, projections of efficacy, additional/standard treatment options must be weighed into decisions to advance clinically with such a profile for this scenario.

**FIGURE 2:**
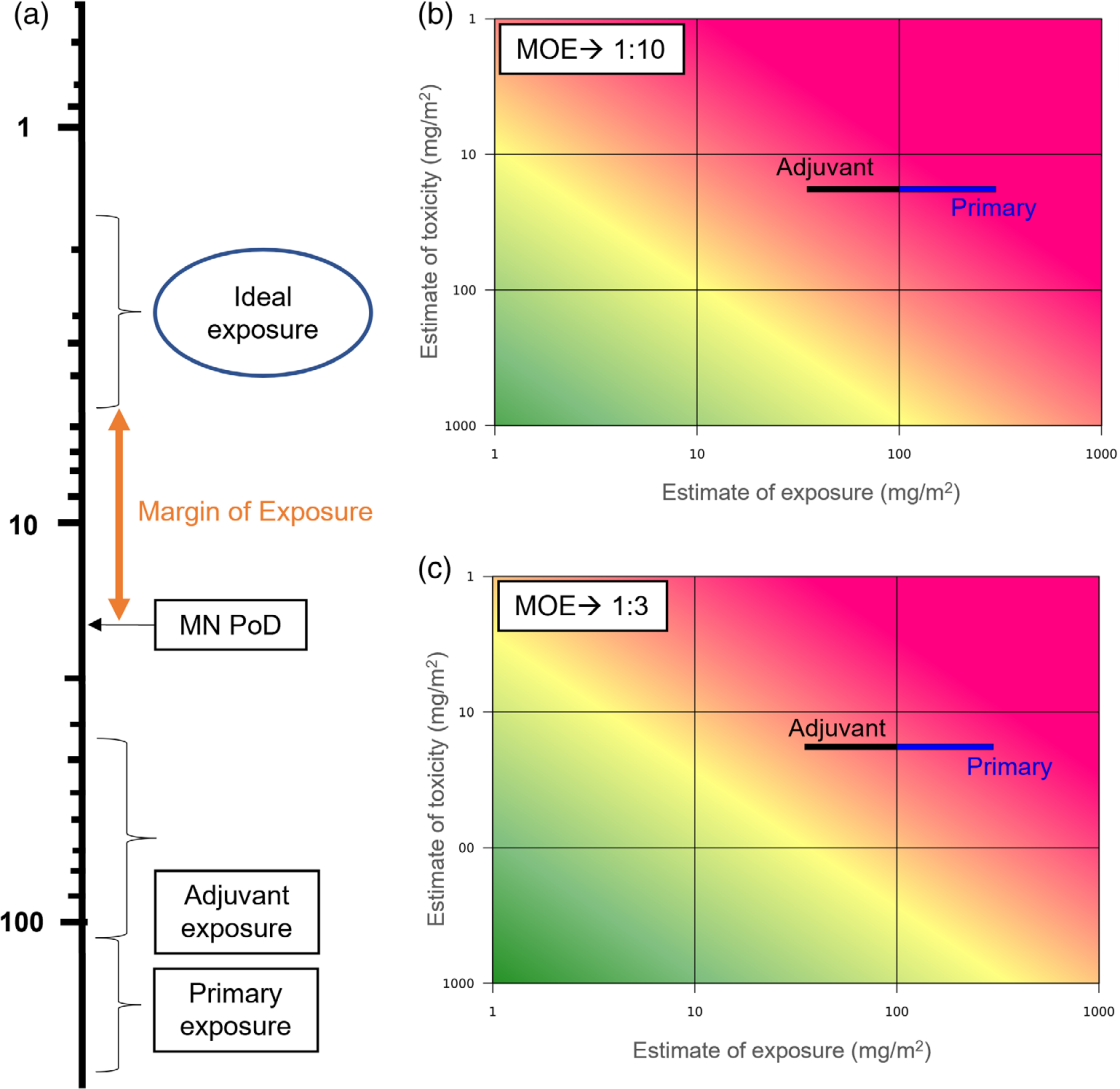
Diagrammatic presentations of the calculated margins of exposure (MOEs) for the hypothetical etoposide adjuvant therapy exposure case study compared to primary use exposures for etoposide as an adjuvant and primary therapy. The MOE in each presentation is based on the PoD calculated from the in vivo micronucleus study discussed in the text (2.89 mg/kg or 17.4 mg/m^2^). Units are in mg/m^2^ for all diagrams. (a) is a linear presentation. (b) and (c) are based on plots from Embry et al. (2014); see publication for more detail on plot generation. The MOE range is shown as a 1:10 ratio (b) and as a 1:3 ratio (c). The green area represents a more acceptable MOE, the yellow a borderline acceptable/unacceptable MOE, and the red a less desirable MOE

In this hypothetical case study based on exposure levels consistent with adjuvant therapy for cancer patients, the target would be a sufficient MOE to predict low risk (as seen in Figure 2a). When nonclinical toxicities are observed, the hope is that an adequate safety margin, that is, MOE, can be established in the clinical trial. While there are no defined safety margin requirements for various treatment indications, pharmaceutical sponsors typically look for at least a 10‐fold margin for non‐oncology indications. Due to the life‐threatening nature of cancer, margins under 1 can be acceptable under certain circumstances (ICH, 2009b).

For this hypothetical case, an acceptable MOE range of 3‐ to 10‐fold was targeted (Figure 2) using 2.89 mg/kg (17.4 mg/m^2^) as the POD in relation to a 35–100 mg/m^2^ treatment, representing a range of typical etoposide doses but limited for the purposes of this case example. The plots in B and C of Figure 2 show that the range of etoposide exposures is probably not large enough (ranges falling in the red zone) to ensure an adequate margin to safely expose people either for a clinical trial or for intended therapy under this scenario. However, it can be pointed out to decision makers that the dose(s) intended for this scenario are not far from a possible “acceptable” dose range (yellow zone) and are under dosing regimens seen with actual etoposide use in cancer therapies. In this simplified case study, working from the assumption that an adequate MOE would be needed for the intended population, the likely outcome from the risk characterization based solely on genetic toxicity would be that the proposed use of etoposide would not be generally appropriate. However, risk managers/decision makers would likely take other factors into account, especially benefits to a population that needs therapy, and then could decide differently.

## 3 EVALUATION OF ETOPOSIDE AS A PHARMACEUTICAL CASE STUDY—CONCLUSIONS

The use of etoposide as a hypothetical case study was insightful. The initial aim was to retrospectively apply the framework and identify adjustments that could be specifically incorporated for pharmaceutical applications. Upon review, it became apparent that much of the framework is already routinely applied during the genotoxicity assessment of pharmaceutical agents. A drug candidate may cause direct DNA damage and poses a risk to healthy volunteers or patients, so this concern must be addressed early in discovery and/or development phases (planning and scoping); knowledge of the drug target and possible off‐targets effects, structural moieties and reactivity, exposure and distribution, and metabolism are typically gathered to determine if there could be potential for genetic damage (e.g., for small molecules, systemically available drugs) or not (e.g., for biological drugs, small molecules not absorbed) (building a knowledge base).

While the framework does not radically alter the existing pharmaceutical industry practices, it does offer a systematic and comprehensive evaluation approach and provides a path for quantitation of risk. The framework additionally prompts the user to consider a broader array of testing and information tools beyond the traditional genetic toxicology test battery approach.

By scoping and planning what data are needed, that is, either negative results for genotoxicity or an acceptable MOE, we showed how the framework helped us decide the *appropriate* testing for the specific clinical situation as opposed to a default approach for drugs intended for treatment of cancer patients. As existing data indicated possible risks for mutation or chromosome damage (Table 3), appropriate in vivo studies were identified to address patient risk. An important learning was that while current regulatory guidelines provide suggestions for in vivo testing and study design to support qualitative conclusions, few of the etoposide literature studies were designed to generate robust dose–response data. While existing regulatory test data may be incorporated into the framework, a fundamental shift in study objectives—to obtain quality data to inform a dose–response analysis—needs to be incorporated. Although current testing guidelines aim to identify genotoxic hazards, this framework encourages study designs that result in dose–response data that can be used for quantitative analysis and derivation of genotoxic PODs. Risk characterization and a risk/benefit profile encompassing acceptable exposure safety multiples may then be applied to enable human dosing of genotoxic drugs, at dose levels that impart acceptable risk/benefit.

While some genotoxicity assays were originally designed for hazard identification, with the proper dose response data, quantitative analysis of such data sets is possible. For example, both Fiedler et al. (2010) and Garriott et al. (1995) explored dosages of 57 mg/kg, with Fiedler employing doses above and below (114 mg/kg as high dose, with dose declinations of 50%) given orally for 2 consecutive days. Garriott et al. included a micronucleus assessment on a 14‐day study with a high dose of 57 mg/kg along with doses of 11.36 and 1.4 mg/kg. MN‐PCE responses to etoposide treatment in the Garriott study provided a strong dose response (0.1%–8.7% MN‐PCE in male rats; 0.14%–1.86% MN‐PCE in female rats) and provided greater confidence in the BMD calculation with tighter CIs (Table S1, Figure 1) and was subsequently used in our risk assessment. Data from Fiedler et al. (Table S2) was assessed for the BMD (Fiedler et al., 2010) however, the dose response for MN% was only within the medium range (2.3%–4.9% across the dose range of 14.3–114 mg/kg), not covering the low or high response regions, and therefore did not provide precise BMD CIs, as shown by higher BMD CI ratios (Table S3; Figure S1) (Fiedler et al., 2010).

While the review of studies indicated that determination of a more precise BMD requires a robust dose response, BMD may be determined from a standard in vivo micronucleus design, provided that an ample range and number of dose levels are included. This observation emphasizes the importance of the initial planning and scoping steps, in which proactive consideration for the type of data needed to solve the problem must be identified to select the appropriate assay and study design. While the Garriott et al. (1995) experiments were not intended to identify a PoD, the results were amenable to such an analysis. In vivo genotoxicity studies that offer an appropriate number of dosing groups and/or target plasma exposures that can be compared to projected efficacious plasma levels may help provide answers to the problem statement.

In our case study, BMD_50_ ranges were derived. Human dose equivalents imparting an acceptable level of genotoxic risk, which still imparted efficacious anticancer response, would then be derived. Assessment of pharmaceutical impurities is an alternative case study that could produce high BMD_50_ values which could be controlled by setting low specification levels. As illustrated by these different scenarios, in the planning and scoping step, it is important to consider possible outcomes and the critical problem statement.

While this exercise was not intended to justify use of etoposide monotherapy and/or represent a real‐life clinical indication, this case study demonstrated the steps and outcomes of this framework for examining genetic damage with etoposide as a model drug and was an insightful effort which helped identify the key data among the wealth of available existing data. Etoposide's well‐understood mechanism of action as a topoisomerase II inhibitor, and the positive genotoxicity results covering multiple doses both in vitro and in vivo, allowed for derivation of a benchmark dose. Clarification of the targeted population during planning and scoping was determined to be crucial step to select/design studies and produce data that informed risk/benefit for the intended patient population.

## CONFLICT OF INTEREST

The authors declare no conflict of interest.

## AUTHOR CONTRIBUTIONS

John Nicolette, Kerry L. Dearfield, Jennifer C. Sasaki, and Mirjam Luijten provided leadership to the GTTC working group leading this case example and led the preparation and writing of the manuscript. Laura Custer, Gladys Ouedraogo, Roland Froetschl, George Johnson, Raja Settivari, and Veronique Thybaud all provided written contributions pertaining to their expertise and participated in the preparation and editing of the manuscript. Michelle Embry participated in the preparation of figures and editing of the manuscript and provided logistical, organizational, and editorial support for the project. All authors approved the final manuscript.

## Supporting information


**Table S1** Bone marrow polychromatic erythrocytes (PCE) micronuclei (MN) percentages from (Garriott et al., 1995), 14‐day oral gavage study in Fischer 344 rats

**Table S2** Bone marrow polychromatic erythrocytes (PCE) micronuclei (MN) percentages from (Fiedler et al., 2010), oral gavage dosing for 2 days in Sprague–Dawley rats

**Table S3** Covariate BMD analysis using a CES of 50% was carried out using PROAST v65.5. Dose response data from the MN PCE% in male rats was assessed from the Fiedler 2010 publication (Fiedler et al., 2010). The lowest BMDL and highest BMDU from the Hill and exponential models (Figure S1) are presented

**Figure S1** BMD analysis of dose response data from the MN PCE% in male rats from the (Fiedler et al., 2010) publication, using a CES of 50%. Results are shown for the exponential and Hill models. Log10 used for each axis
