# Supplementary material for: Utility of a next‐generation framework for assessment of genomic damage: A case study using the pharmaceutical drug candidate etoposide
Source: Environ Mol Mutagen. 2021 Nov 22;62(9):512–25. doi: 10.1002/em.22467 (PMC9299499; doi:10.1002/em.22467)
Supplement: Supplementary file 1 — Table S1 Bone marrow polychromatic erythrocytes (PCE) micronuclei (MN) percentages from (Garriott et al., 1995), 14‐day oral gavage study in Fischer 344 rats [file EM-62-512-s002.docx]

**Supplementary Table S1:** Bone marrow polychromatic erythrocytes (PCE) micronuclei (MN) percentages from (Garriott et al. 1995), 14-day oral gavage study in Fischer 344 rats

| Sex | Dose (mg/kg/day) | Total dose (mg/kg) | HED (Human Equivalent Dose, mg/m^2^/day)^a^ | Mean MN% | SD^b^ | N per group |
| --- | --- | --- | --- | --- | --- | --- |
| Male | 0 | 0 | 0 | 0.08 | 0.08 | 5 |
| Male | 1.14 | 15.96 | 6.8 | 0.1 | 0.07 | 5 |
| Male | 11.36 | 159.04 | 68.0 | 0.34 | 0.21 | 5 |
| Male | 57 | 798 | 340 | 8.72 | 1.78 | 5 |
| Female | 0 | 0 | 0 | 0.1 | 0.1 | 5 |
| Female | 1.14 | 15.96 | 6.8 | 0.14 | 0.11 | 5 |
| Female | 11.36 | 159.04 | 68.0 | 0.24 | 0.25 | 5 |
| Female | 57 | 798 | 340 | 1.86 | 0.42 | 5 |

^a^ HED (Human Equivalent Dose) calculated per “Guidance for Industry: Estimating the Maximum Safe Starting Dose in Initial Clinical Trials for Therapeutics in Adult Healthy Volunteers. US FDA, CDER, July 2005 (USFDA 2005).

^b^SD, standard deviation.
