# Supplementary material for: Utility of a next‐generation framework for assessment of genomic damage: A case study using the pharmaceutical drug candidate etoposide
Source: Environ Mol Mutagen. 2021 Nov 22;62(9):512–25. doi: 10.1002/em.22467 (PMC9299499; doi:10.1002/em.22467)
Supplement: Supplementary file 2 — Table S2 Bone marrow polychromatic erythrocytes (PCE) micronuclei (MN) percentages from (Fiedler et al., 2010), oral gavage dosing for 2 days in Sprague–Dawley rats [file EM-62-512-s001.docx]

**Supplementary Table S2:** Bone marrow polychromatic erythrocytes (PCE) micronuclei (MN) percentages from (Fiedler et al. 2010), oral gavage dosing for 2 days in Sprague-Dawley rats

| Sex | Dose (mg/kg/day) | Total dose (mg/kg) | HED (Human Equivalent Dose, mg/m^2^/day)^a^ | Mean MN% | SD^b^ | N per group |
| --- | --- | --- | --- | --- | --- | --- |
| Male | 0 | 0 | 0 | 0.38 | 0.09 | 4 |
| Male | 14.25 | 28.5 | 86.7 | 1.28 | 0.26 | 4 |
| Male | 28.5 | 57 | 170 | 1.61 | 0.47 | 4 |
| Male | 57 | 114 | 340 | 2.12 | 0.58 | 4 |
| Male | 114 | 228 | 680 | 3.37 | 1.83 | 4 |
