# Supplementary material for: Utility of a next‐generation framework for assessment of genomic damage: A case study using the pharmaceutical drug candidate etoposide
Source: Environ Mol Mutagen. 2021 Nov 22;62(9):512–25. doi: 10.1002/em.22467 (PMC9299499; doi:10.1002/em.22467)
Supplement: Supplementary file 3 — Table S3 Covariate BMD analysis using a CES of 50% was carried out using PROAST v65.5. Dose response data from the MN PCE% in male rats was assessed from the Fiedler 2010 publication (Fiedler et al., 2010). The lowest BMDL and highest BMDU from the Hill and exponential models (Figure S1) are presented [file EM-62-512-s004.docx]

**Supplementary Table S3**: Covariate BMD analysis using a CES of 50% was carried out using PROAST v65.5. Dose response data from the MN PCE% in male rats was assessed from the Fiedler 2010 publication (Fiedler et al. 2010). The lowest BMDL and highest BMDU from the Hill and exponential models (Supplementary Figure S1) are presented.

| **BMD Confidence interval bounds** | **MN PCE% Flow**  **Exponential** | **MN PCE%**  **Flow**  **Hill** | **MN PCE%**  **Microscopy**  **Exponential** | **MN PCE%**  **Microscopy**  **Hill** |
| --- | --- | --- | --- | --- |
| *BMDL_50_* (mg/kg)  (CES 50%) | *0.039* | *0.0137* | *5.16x10^-06^* | *1.56x10^-06^* |
| *BMDU_50_* (mg/kg)  (CES 50%) | *3.16* | *3.25* | *9.84* | *0.000156* |
| *BMDL_50_: BMDU_50_ ratio* | *81* | *237* | *1,906,976* | *100* |

*BMDL: lower confidence limit of BMD; BMDU: upper confidence limit of BMD*
