# Supplementary figures and images for: Utility of a next‐generation framework for assessment of genomic damage: A case study using the pharmaceutical drug candidate etoposide
Source: Environ Mol Mutagen. 2021 Nov 22;62(9):512–25. doi: 10.1002/em.22467 (PMC9299499; doi:10.1002/em.22467)

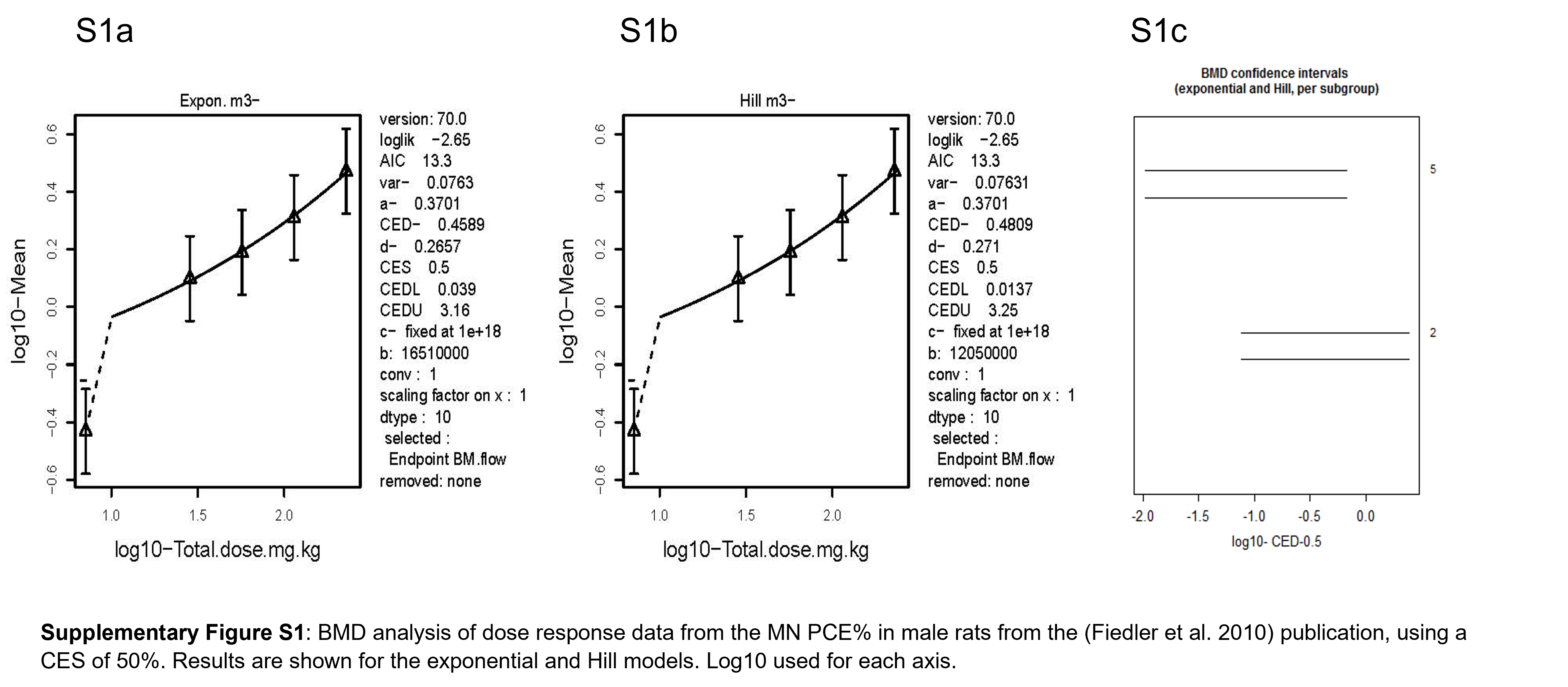

Supplement: Supplementary file 4 — Figure S1 BMD analysis of dose response data from the MN PCE% in male rats from the (Fiedler et al., 2010) publication, using a CES of 50%. Results are shown for the exponential and Hill models. Log10 used for each axis [file EM-62-512-s003.tif]
